# Application of Immune Checkpoint Inhibitors in Gynecological Cancers: What Do Gynecologists Need to Know before Using Immune Checkpoint Inhibitors?

**DOI:** 10.3390/ijms24020974

**Published:** 2023-01-04

**Authors:** Seon-Mi Lee, Sanghoon Lee, Hyun-Woong Cho, Kyung-Jin Min, Jin-Hwa Hong, Jae-Yun Song, Jae-Kwan Lee, Nak-Woo Lee

**Affiliations:** 1 Department of Obstetrics and Gynecology, Korea University College of Medicine, 73 Koreadae-ro, Seongbuk-gu, Seoul 02841, Republic of Korea; 2 Department of Obstetrics and Gynecology, Korea University College of Medicine, 148, Gurodong-ro, Guro-gu, Seoul 08308, Republic of Korea; 3 Department of Obstetrics and Gynecology, Korea University College of Medicine, 123, Jeokgeum-ro, Danwon-gu, Ansan-si 15355, Gyeonggi-do, Republic of Korea

**Keywords:** immune checkpoint inhibitor therapy, gynecological cancer, immune-related adverse events

## Abstract

Standard treatments for gynecological cancers include surgery, chemotherapy, and radiation therapy. However, there are limitations associated with the chemotherapeutic drugs used to treat advanced and recurrent gynecological cancers, and it is difficult to identify additional treatments. Therefore, immune checkpoint inhibitor (ICI) therapy products, including PD-1/PD-L1 inhibitors and CTLA-4 inhibitors, are in the spotlight as alternatives for the treatment of advanced gynecological cancers. Although the ICI monotherapy response rate in gynecological cancers is lower than that in melanoma or non-small cell lung cancer, the response rates are approximately 13–52%, 7–22%, and 4–17% for endometrial, ovarian, and cervical cancers, respectively. Several studies are being conducted to compare the outcomes of combining ICI therapy with chemotherapy, radiation therapy, and antiangiogenesis agents. Therefore, it is critical to determine the mechanism underlying ICI therapy-mediated anti-tumor activity and its application in gynecological cancers. Additionally, understanding the possible immune-related adverse events induced post-immunotherapy, as well as the appropriate management of diagnosis and treatment, are necessary to create a quality environment for immunotherapy in patients with gynecological cancers. Therefore, in this review, we summarize the ICI mechanisms, ICIs applied to gynecological cancers, and appropriate diagnosis and treatment of immune-related side effects to help gynecologists treat gynecological cancers using immunotherapy.

## 1. Introduction

The statistics conducted by the World Cancer Research Fund International (WCRF International) identified cervical cancer as the fourth most newly diagnosed cancer in women and the seventh most common cancer among all cancers. More than 604,000 patients were newly diagnosed with cervical cancer in 2020 [1]. Endometrial cancer is the sixth most common cancer in women and the fifteenth most common cancer among all cancers. In 2020, more than 417,000 newly diagnosed endometrial cancer patients were reported [2]. Ovarian cancer is the eighth most common cancer in women and the eighteenth most common cancer among all cancers. The number of newly diagnosed ovarian cancer patients in 2020 was estimated to be more than 313,000 [3]. The relative five-year survival rates of patients with gynecological cancers from 2012 to 2018 were reported to be 66.7%, 81.3%, and 49.7% for cervical, endometrial, and ovarian cancers, respectively [4,5,6]. According to the statistics reported by the NIC (National Cancer Institute), the proportion of patients dying from cervical cancer has gradually decreased from a 3.5 rate per 100,000 people in 1992 to a 2.2 rate per 100,000 people in 2019 [4]. The mortality rate of endometrial cancer patients increased slightly from a 4.2 rate per 100,000 people in 1992 to a 5.0 rate per 100,000 people in 2019 [5] and remained the same thereafter. The mortality rate of ovarian cancer patients decreased from a 9.5 rate per 100,000 people in 1992 to a 6.2 rate per 100,000 people in 2019 [6].

Although the mortality rate of gynecological cancer patients is decreasing, due to the development of surgical and diagnostic technologies and anticancer drugs, a considerable amount of effort is essential to increase the survival rate of patients. In clinical practice, adjuvant chemotherapy alone is often insufficient for treating patients with advanced-stage gynecological cancers. Immune checkpoint inhibitor (ICI) therapy was developed to overcome these limitations, and representative examples include PD-1, PD-L1, and CTLA-4 inhibitors. PD-1 expressed in T cells or antigen-presenting cells (APCs) interacts with the PD-L1 expressed in tumor cells, resulting in the inhibition of intracellular signaling and effector T cell activation; thus, PD-1 functions as a checkpoint in the immune process [7]. As a result of the interaction between T cell’s CTLA-4 and B7 expressed in APC, the immune response to tumor cell is suppressed, and CTLA-4 also functions as a checkpoint in the immune process [7]. Previous studies have reported that pembrolizumab and nivolumab, which belong to PD-1 inhibitors, perform tumor suppressive functions in non-small cell lung cancer (NSCLC) and melanoma. Anti-PD-L1 agents, such as atezolizumab, durvalumab, and avelumab, are also reported to have anti-tumor effects in several cancers [8,9]. However, these immunotherapies can cause immunotolerance imbalance, resulting in immune-related adverse events (irAEs). IrAEs are autoimmune conditions that can affect any organ in the whole body after ICI therapy. IrAEs can be mild skin rashes, itching sensations, gastrointestinal diseases, endocrine adverse events, and serious adverse events that threaten life, such as myasthenia gravis and myocarditis.

Therefore, it is critical to understand the consequences of ICI use and to manage them. In this review, we aimed to summarize the mechanisms of actions of various ICIs, namely PD-1 inhibitors, PD-L1 inhibitors, and anti-CTLA-4 antibodies; mechanisms that cause irAEs; cases of ICI use in gynecological cancer; and clinical symptoms and appropriate management of various irAEs.

## 2. PD-1/PD-L1 Inhibitor Mechanism

**Figure 1:**
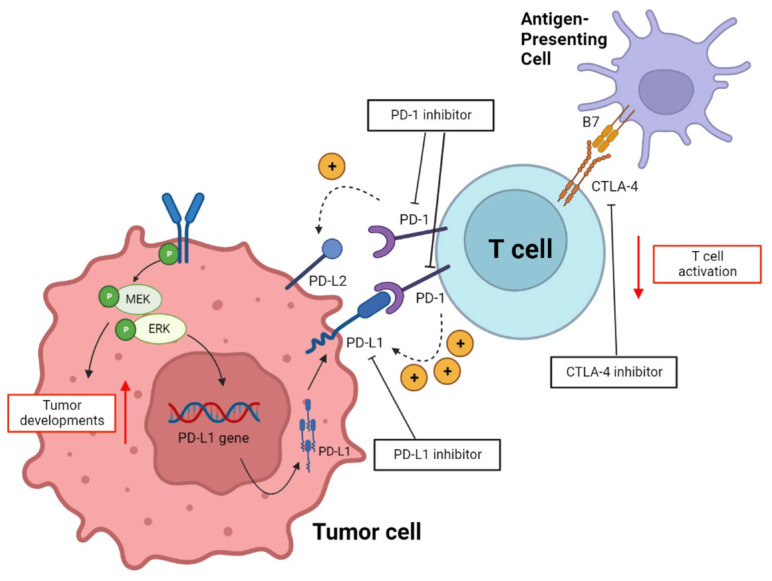
Interaction between PD-1/PD-L1 and CTLA-4/B7 in the immune system and mechanism expression of PD-1/PD-L1 inhibitor and CTLA-4 inhibitor.

PD-1 is a type 1 transmembrane protein belonging to the immunoglobulin superfamily, also called CD27 [10]. PD-1, a well-known checkpoint for T lymphocyte-related immune processes, is expressed in T cells, as well as in antigen-presenting cells (APCs), such as B cells, natural killer T cells, dendritic cells, and macrophages [11]. PD-1 can interact with PD-L1 and PD-L2 ligands; however, the affinity with PD-L1 appears to be approximately three times higher than that of PD-L2 [7]. PD-L1 and PD-L2 are membrane proteins that are expressed in tumor cells and APCs. Toll-like receptor (TLR)-mediated signaling is required for PD-L1 expression [12]. The signaling transmission system of TLR is activated by MEK/ERK kinase and functions as cell cycle regulators, and an unregulated MEK/ERK kinase pathway can induce the tumor developments, which activates the transcription of PD-L1 mRNA, resulting in the expression of PD-L1 as a membrane protein of the tumor cell [12]. The interaction between PD-L1 expressed in tumor cells and PD-1 expressed in T cells inhibits the activity of effector T cells and increases the secretion of proinflammatory cytokines, such as tumor necrosis factor (TNF)-alpha, interleukin (IL)-2, and interferon-gamma (IFN-γ) [13]. Among the secreted cytokines, IFN-γ receptors 1 and 2 induce JAK/STAT-mediated IRF-1 activation, increasing the expression of PD-L1 in regulatory T cells, tumor-associated macrophages, and myeloid-derived suppressor cells within the tumor microenvironment [13]; as a result, the immunosuppressive microenvironment in a high state is maintained, and the aggressiveness of the tumor cell is accelerated. Considering that the immunomodulatory ability of PD-1/PD-L1 occurs at the tumor site or peripheral tissue, anti-PD-1/PD-L1 antibody agents are used to inhibit tumor cell growth by activating effector T cells, regulatory T cells, and B cells in the late T cell-mediated immune response [14]. The interaction between PD-1 and PD-L1, introduced above, is expressed in Figure 1.

## 3. CTLA-4 Inhibitor Mechanism

CTLA-4 is one of 28 families of mammalian proteins activated in naïve T cells [15]. The ligand for CTLA-4 is B7, which is expressed in APCs. Their interaction occurs at the lymph node, during the initial stage of the immune response, and when the B7 in the APC recognizing the tumor cell reacts with CTLA-4 in T cells; as a result, the immune response against tumor cells is suppressed [7,16]. Although CTLA-4 and PD-1/PD-L1 play a similar role in inhibiting the immune response against tumor cells, their timing of activation in the immune phase differs; CTLA-4 performs immune response regulation as a checkpoint in the early stages of immunity and at the lymph nodes, whereas PD-1 acts during the late stage of the immune response. Previous studies comparing the immunological activity in CTLA-4 gene knockout mice and PD-1 gene knockout mice [17,18] demonstrated that, in CTLA-4 gene knockout mice, T cell blasts stimulated by upregulated activation markers accumulated, leading to severe lymphoproliferative diseases that generally affect the liver, heart, lungs, and pancreas [17], whereas in PD-1 knockout mice, organ-specific toxicity similar to graft versus host disease was observed [18]. The interaction between CTLA-4 in T cell and B7 in APC, described above, is represented in Figure 1.

PD-1 in T cells can interact with PD-L1 or PD-L2 in tumor cells, but the affinity between PD-1 and PD-L1 is stronger than the affinity between PD-1 and PD-L2. As a result of the interaction between PD-1 and PD-L1, MEK/ERK kinase in tumor cells is activated, and PD-L1 expression in tumor cells is increased, as well as tumor cell development. On the other hand, the activity of the T cells is inhibited, and as a result of the interaction between CTLA-4 of T cells and B7 of APC, T cell activity is also inhibited. In this mechanism, the PD-1 inhibitor, PD-L1 inhibitor, and CTLA-4 inhibitor contribute to suppressing tumor cell development and increasing T cell activity.

## 4. Use of ICIs in Endometrial Cancer

Endometrial cancer is classified into the following four categories, according to molecular genetic analysis using The Cancer Genome Atlas (TCGA) database: DNA polymerase epsilon (DNA-POLE), microsatellite instability-high (MSI-H), copy number high (CNH; p53 abn), and copy number low (CNL; p53 wt) [19]. The POLE mutation has an ultra-mutated DNA sequence, which is well-recognized by the immune system and has a good prognosis in high-risk endometrial cancer patients [20]. Patients in the MSI-H group have an intermediate prognosis. Among CNH and CNL, which are distinguished by the presence or absence of p53 mutation, CNH, whose function is either overexpressed or lacks function due to p53 alteration, causes missense mutations and has the worst prognosis. Endometrial cancers of the p53 abnormal molecular type are often serous and mixed types, high stage, Grade 3 or higher as a result of biopsy [21]. Among these molecular types of endometrial cancer, the MSI-H group responds well to immunotherapy. In a mismatch repair-deficient (MMR-d) environment with a deficient, at least one of the following is involved in mismatch repair (MMR): MutL homolog 1 (MLH1), MutS protein homolog 2 (MSH2), MutS homolog 6 (MSH6), and PMS1 homolog 2 (PMS2); if there is a problem in the DNA strand repair, the occurrence of MSI-H increases even more, so it can be said that MSI and MMR-d are related to each other [22]. MMR-d tumors can result from somatic mutations in MMR genes or from Lynch syndrome that causes congenital mutations, and the probabilities are as follows: among all endometrial cancers, the MMR-d group accounts for approximately 23–36%, of which the MMR-d group induced by Lynch syndrome accounts for approximately 2% [21]. In MSI-H and MMR-d environments, the occurrence of neoantigens is further increased to activate the immune activity in the body [22]. In a study performed by Xiao et al., wherein the degree of MMR-d was compared with the degree of MSI-H, it was observed that MSI-H increased further in the MMR-d environment [23]. In addition, when a PD-1 inhibitor was used in the MMR-d tumor cell environment, the CD8^+^ T cell activity was promoted, compared to that in the mismatch-proficient (MMR-p) tumor cell environment, in which all MMR proteins, including MLH1, MSH2, MSH6, and PMS2, were expressed and tumor cell apoptosis was further increased [24]. Based on these principles, many studies have reported the results of ICI use in clinical practice. The results of previous studies, which were conducted to improve the therapeutic efficacy, using a combination of immunotherapy and anti-angiogenesis agents or chemotherapy, as well as ICI monotherapy, are summarized in Table 1. The phase 2 study using a PD-1 inhibitor between the MMR-d tumor and MMR-p tumor showed that, in the MMR-d tumor, the objective response rate (ORR) was 40% and the progression-free survival (PFS) rate at 20 weeks was 67%, and these were significantly higher than those in the MMR-p tumor (*p* = 0.02) in the case of endometrial cancers. The MMR-d colorectal cancer group also reported a higher ORR and PFS rate at 20 weeks than the MMR-p colorectal cancer group (MMR-d colorectal cancer group: ORR 40%, PFS rate at 20 weeks 78%; MMR-p colorectal cancer group: ORR 0%, PFS rate at 20 weeks 11%) [25]. KEYNOTE 016, 158, 028 studies analyzed the outcome when pembrolizumab was not used in endometrial cancer patients with MMR-d/MSI-H molecular characteristics. The results demonstrated that the MMR-d/MSI-H group had a complete response rate of 7.4% and a partial response rate of 32.2%, and it was reported to have an effect with an ORR of 39.6% [26]. The above studies demonstrated that PD-1 inhibitors are more effective when there is a molecular characteristic of MMR-d or MSI-H among endometrial cancer patients.

**Table 1:** ICI monotherapy and Combination therapy, including ICI applied to endometrial cancer.

Studies	Patient Subjects	Therapeutic Agent	Results
Studies using single ICI therapy			
Phase 2 study conducted by Le et al. (NCT01876511) [25]	EM cancer with MMR-d (EM cancer 2 out of 9 MMR-d non-colorectal cancer patients)	Pembrolizumab	ORR, 40%
Multicohort phase Ib study conducted by Ott et al. (KEYNOTE-028 study) [27]	Advanced or metastatic EM cancer with PD-L1-positive	Pembrolizumab	ORR, 13%
KEYNOTE 016, 158, 028 [26]	EM cancer with MMR-d/MSI-H (EM cancer 14 out of 59 MMR-d/MSI-H non-colorectal cancer patients	Pembrolizumab	ORR, 39.6%
Study conducted by Santin et al. [28]	2 patients with EM cancer (POLE and MSI-H)	Nivolumab	Prolonged response for more than 7 months in 2 patients
Phase 2 study conducted by Hasegawa et al. [29]	23 patients with metastatic EM cancer	Nivolumab	ORR, 23% PFS, 3.6 months
Phase Ia study conducted by Fleming et al. [30]	15 patients with metastatic EM cancer	Atezolizumab	ORR, 13% PFS, 1.7 months
Phase I/II GARNET trial conducted by Oaknin et al. [31]	Advanced/recurrent EM cancer with MSI-H	TSR-042	ORR, 52%
Studies using combination therapy (ICI + antiangiogenesis agent)			
Phase Ib/II study conducted by Makker et al. (KEYNOTE 775) [32]	Metastatic EM cancer	Pembrolizumab + Lenvatinib	ORR, 48% DCR, 96%
Phase II study Conducted by Moore et al. (NCT03526432) [33]	Recurrent EM cancer	Atezolizumab + Bevacizumab	Ongoing
Phase II study conducted by Lheureux et al. (NCT03367741) [34]	Recurrent EM cancer	Nivolumab + Cabozantinib	ORR, 25% PFS 5.3 months (MSI-H) Clinical benefit (ORR+SD) higher than nivolumab single therapy group; *p* < 0.001
Studies using combination therapy (ICI + chemotherapy)			
Phase II study conducted by Matei et al. (NCT02549209) [35]	Advanced/recurrent EM cancer	Pembrolizumab + Chemotherapy (Paclitaxel and Carboplatin)	Ongoing
Phase II study conducted by Vall d’Hebron Institute of Oncology (NCT03276013) [36]	Recurrent/metastatic EM cancer	Pembrolizumab + Chemotherapy (Doxorubicin)	Ongoing
Phase III study conducted by Colombo et al. (NCT03603184) [37]	Advanced/recurrent EM cancer	Pembrolizumab + Chemotherapy (Paclitaxel and Carboplatin)	Ongoing
Phase II study conducted by Pignata et al. (NCT03503786) [38]	Advanced/recurrent EM cancer	Avelumab + Chemotherapy (Paclitaxel and Carboplatin)	Ongoing

EM, endometrial; ICI, immune checkpoint inhibitor; MMR-d, mismatch repair protein deficiency; MSI-H, microsatellite instability-high; POLE, polymerase-epsilon; ORR, objective response rate; PFS, progression-free survival; DCR, disease control rate; SD, stable disease.

The KEYNOTE-028 multicohort study reported that the use of pembrolizumab in treating advanced or metastatic endometrial cancer patients resulted in an ORR of 13% in PD-L1-positive patients. This demonstrated that, in patients with PD-L1-positive adverse or metastatic endometrial cancer, pembrolizumab is effective, as well as less risky, to use [27]. In other words, the use of PD-1 inhibitors in endometrial cancer patients with MMR-d helps increase efficacy, and although severe sequelae that require discontinuation of the drug did not occur, mild sequelae might occur. Studies such as NCT0352643 (immunotherapy + antiangiogenesis agent), NCT02549209 (immunotherapy +chemotherapy), NCT03276013 (immunotherapy + chemotherapy), NCT03603184 (immunotherapy + chemotherapy), and NCT 03503786 (immunotherapy + chemotherapy), which combine immunotherapy with chemotherapy or angiogenesis suppressive therapy, are currently underway and aim to increase the treatment effectiveness in advanced endometrial cancer [33,35,36,37,38]. In KEYNOTE 775, a phase Ib/II study conducted by Makker et al., the ORR and disease control rate (DCR) were evaluated after using lenvatinib 20mg/day plus pembrolizumab 200mg in patients with metastatic endometrial cancer [32]. DCR was defined as the complete response (CR) + partial response (PR) + stable disease (SD), and as a result, promising results were confirmed at 48% (all PR) of ORR and 74% of DCR [32]. In the NCT03367741 study, conducted by Lheureux et al., the ORR and PFS were compared between the nivolumab plus cabozantinib (anti-angiogenesis agent) group and the group using nivolumab [34]. The nivolumab plus cabozantinib group included 36 people, and the nivolumab groups included 18 people, whose PFS and OS are as follows (nivolumab plus and cabozantinib; PFS, 5.3 months; ORR, 25%), (nivolumab; PFS, 1.9 months; ORR 16.7%). Clinical benefits were compared through ORR + SD in theses two groups, and as a result, the group using nivolumab + carbozantinib was significantly higher than the group using nivolumab (nivolumab plus and cabozantinib; clinical benefits, 67.4%) (nivolumab; clinical benefits, 27.8%) [34].

## 5. Use of ICIs in Ovarian Cancer

When ovarian cancer is initiated, an immune response occurs as a result of tumor-infiltrating lymphocytes (TILs) activation. Several papers have been published to identify positive or negative relationships between the number and type of TILs and ovarian cancer treatment [39,40,41]. According to a study conducted by Zhang et al. to evaluate the association between CD3^+^ TIL and outcomes in ovarian cancer intratumoral T cells, the five-year overall survival (OS) of the patient group with TIL in tumor cell islets was 38.0%, and this was significantly higher than the five-year OS in the patient group without TIL [39]. In addition, the five-year survival rate after receiving platinum-based chemotherapy post-debulking surgery was 73.9% in patients with tumors containing TILs, and this was higher than the five-year survival rate in patients with tumors that did not contain TILs (11.9%) [39]. Multivariate analysis showed that IFN-γ, IL-2, and lymphocyte-attracting chemokines increased within tumor cells in the presence of TILs, demonstrating a meaningful association with delayed tumor cell recurrence or death. However, it has been reported that, in the absence of TILs in tumor cells, vascular endothelial growth factor increases, thereby contributing to tumor growth [39]. In another study, there was no association between CD3^+^ TILs and outcomes in ovarian cancer patients, but the survival period in groups with a high CD8^+^/CD4^+^ ratio was longer than that in groups with low CD8^+^/CD4^+^ ratio (high CD8^+^/CD4^+^ ratio survival period, 74 months; low CD8^+^/CD4^+^ ratio survival length, 25 months; hazard ratio = 0.31, *p* = 0.0002) [40]. Owing to this, it was suggested that the CD8^+^/CD4^+^ (Treg) ratio is associated with a favorable prognosis in epithelial ovarian cancer. In contrast, a study reported that there is a negative correlation between the regulatory T cell activity of CD4^+^ and CD25^+^ and the outcome of ovarian cancer patients and that CD4^+^CD25^+^FOXP3^+^Treg cells tend to accumulate primarily in tumors or ascites. When the chemokine CCL22, produced by macrophages, as well as tumor cells of ovarian cancer, is secreted, regulatory T cells are further clustered to suppress T cell-mediated immune response against tumor cells, thus creating an environment that promotes cancer growth [41]. The association between changes in the composition of lymphocytes formed in ovarian cancer and outcomes after treatment has also increased with the application of immunotherapy. Epithelial ovarian cancer is classified into serous, mucinous, endometrioid, clear cell, and transitional cell types. Among them, clear cell type epithelial ovarian cancer has MSI-H characteristics at a rate about 10%, and clear cell type ovarian cancer with MSI-H has a higher expression of PD-1 than serous type ovarian cancer [19]. In addition, clear cell and endometroid ovarian cancers account for a high proportion in Lynch syndrome, which is associated with MMR-d tumors. The expression of PD-1 in the HRD serous ovarian cancer is increased [19]. Although further studies are needed regarding the difference in PD-1 expression according to cell types in ovarian cancer, ICI therapy tended to be more effective in about 10–29% of ovarian cancer, including either MMR-d or MSI-H, associated with Lynch syndrome [19].

In ovarian cancer patients, studies have reported the outcomes of combination therapies of chemotherapeutic drugs, anti-angiogenesis agents, poly ADP ribose polymerase (PARP) inhibitors, immunotherapy, and ICI monotherapy, which are summarized in Table 2. In the KEYNOTE-028 study, it was reported that the use of pembrolizumab is effective in improving antitumor activity in PD-L1-positive patients with advanced ovarian cancer. Since then, a non-randomized multi-cohort study that followed up for 15.5 months after starting pembrolizumab treatment in 26 patients with advanced ovarian cancer has been published. This study reported an ORR of 11.5% (95% CI, 2.4–30.2%), a PFS of 1.9 months (1.8–3.2 months), and an OS of 13.1 months (6.7–17.5 months) [42]. In the NCT02674061 cohort study, patients with advanced and recurrent ovarian cancer were classified as cohort A from the first to the third line and cohort B from the fourth to the sixth line, and each ORR was compared after using pembrolizumab (cohort A—ORR, 7.4%; cohort B—ORR, 9.9%). In addition, the degree of expression of PD-L1 and the responsiveness to pembrolizumab treatment were analyzed, and it was confirmed that the higher the expression of PD-L1, the higher the ORR by pembrolizumab (PDL1 < 1, ORR, 4.1%; PDL1 ≥ 1, ORR, 5.7%; PDL1 ≥ 10, ORR, 10.0%) [43]. Representative characteristics of cancer cells cleared by Weinberg include: (1) continuous cell proliferation; (2) avoidance of growth inhibition; (3) resistance to cell death; (4) permanent replication ability; (5) induction of angiogenesis; and (6) induction of metastasis and infiltration. According to these typical characteristics of cancer cells, there is a limit to inducing cancer cell death by activating the immune response to tumor cells using ICIs in the treatment of the most fatal gynecological cancers. The use of ICIs in actual cancer patients has produced significant clinical benefits in other solid tumors, including melanoma, but the effect in ovarian cancer is modest [44]. Under these conditions, a treatment method using a combination of antiangiogenesis agents and chemotherapy, as well as ICIs, was used as a strategy for treating ovarian cancer. The results of these studies are presented in Table 2. Comparing ORR after ICI monotherapy with that after combination therapy using ICIs with bevacizumab and chemotherapy, showed that the ORR of combination therapy had a higher tendency than that of single therapy. The following studies are currently underway for developing improved treatment strategies: NCT02440425, NCT05116189, NCT02891824, and NCT03596281 [45,46,47,48]. Recently, an anticancer virus has also been developed, and this is one of the cancer treatment methods developed using the characteristics of inducing reinfection in other cells by destroying host cells after the virus penetrates and multiplies in host cells. Various types of antigenic proteins are overexpressed on the surface of cancer cells to help in cell proliferation, and viruses that use these antigens as receptors are reversely used for cancer treatment by using their characteristics. When the virus proliferates in the host cell and subsequently destroys it, the uric acid, adenosine triphosphate (ATP), and heat shock proteins (HSPs) that are released into various cellular contents, as well as virus particles, induce a local inflammatory response. This causes natural killer (NK) cells, dendritic cells (DCs), and macrophages to penetrate cancer cells and sequentially activate T cell-mediated immune reactions to create an environment to attack cancer cells. A study using HSV-1 as a treatment for melanoma reported the characteristics of HSV-1 (herpes simplex virus 1) that recognizes herpesvirus entry mediator (HVEM) overexpressed on the surface of melanoma tumor cells as receptors [49]. Among gynecological cancers, clinical studies have used oncolytic viruses in ovarian, tubal, and peritoneal cancers [50,51]. In a phase I trial study conducted by Galanis et al., the measles virus, Edmonston strain (MV-CEA), was used in the treatment of patients with Taxol and platinum-refractory recurrent ovarian cancer and normal CEA level, resulting in 14 out of 21 subjects’ (approximately 66%) best objective responses, and the total average survival period was 12.15 months (13–38.4 months) [50]. In a randomized phase IIB study for recurrent or persistent ovarian, tubal, or peritoneal cancer, the treatment effect was analyzed by dividing the group into two groups; one group using only weekly paclitaxel and another group using weekly paclitaxel and reolysin virus. The results showed that there was no significant difference in PFS between the weekly paclitaxel group and the combination therapy (weekly paclitaxel + reolysin) group (weekly paclitaxel therapy, PFS 4.3 months; combination therapy, PFS 4.4 months). The ORR was 20.0% for weekly paclitaxel therapy, and this was higher than that of the combination therapy ORR, which was 17.4%. The treatment with reolysin virus in combination with weekly paclitaxel was not helpful in improving recurrence persistent ovarian cancer [52]. However, for the effective treatment of ovarian cancer, studies on novel treatments, such as cancer vaccines, as well as the oncolytic viruses, are underway.

Among the various studies summarized in Table 2, there are studies comparing the results of ICI plus chemotherapy and chemotherapy, which are considered a traditional treatment for ovarian cancer. As a result of the phase III study conducted by Monk et al., the PFS of the ICI plus chemotherapy (CTx.) group was 11 months, slightly longer than the PFS of the CTx. group of 10.2 months [53]. Compared to CTx. group, hazard ratio (HR) for PFS in ICI plus CTx. group was 1.14, but this was not statistically significant as *p* = 0.79 [53]. In the phase III results, performed by Merck et al., the ORR of the avelumab plus CTx. group was 13.3%, higher than the ORR 4.2% of the group that performed CTx. with pegylated liposomal doxorubicin (PLD) [54]. In these studies, in the case of ICI plus CTx., compared to the group using only CTx., no new cellular mechanism was found. However, this is considered to be an issue to be solved through future studies, as well as current studies.

**Table 2:** ICI monotherapy and Combination therapy, including ICI applied to ovarian cancer.

Studies	Patient Subjects	Therapeutic Agent	Results
Studies using single ICI therapy			
Phase I study conducted by Brahmer et al. [55]	Advanced ovarian cancer	Anti-PD-L1 antibody	ORR, 6%
Phase II study conducted by Hamanishi et al. [56]	Platinum resistant ovarian cancer	Nivolumab	ORR, 15% PFS, 3.5 months OS, 20.0 months
Phase Ib study conducted by Disis et al. [57]	Advanced ovarian cancer	Avelumab	ORR, 9.7% PFS, 11.3 weeks OS, 10.8 months
Phase Ia study conducted by Infante et al. [58]	Advanced/recurrent ovarian cancer	Atezolizumab	ORR, 22%
Phase Ib study conducted by Varga et al. (NCT02054806) [42]	PDL1+ advanced ovarian cancer	Pembrolizumab	ORR, 11.5% PFS, 1.9 months OS, 13.1 months
Phase II study conducted by Matulonis et al. (NCT02674061) [43]	Advanced/recurrent ovarian cancer	Pembrolizumab	ORR, 7.4% (one to three prior lines of treatment) ORR, 9.9% (four to six prior lines of treatment)
Studies using combination therapy (ICI + antiangiogenesis agent)			
Phase II study conducted by Liu et al. [59]	Recurrent ovarian cancer	Nivolumab + Bevacizumab	ORR, 21% PFS, 9.4 months
Phase Ib trial conducted by Michels et al. [60]	Platinum resistant ovarian cancer	Pembrolizumab + Bevacizumab	ORR, 26.3%
Studies using combination therapy (ICI + chemotherapy)			
Phase III study conducted by Monk et al. [53]	Ovarian cancer patients who received first line chemotherapy	Avelumab + Chemotherapy (Paclitaxel and Carboplatin)	PFS, 11.0 months (avelumab + CTx.) PFS, 10.2 months (CTx.) HR for PFS, 1.14; 95% CI, 0.83, 1.56; *p* = 0.79 (CTx. = reference)
Phase III study conducted by Merck et al. [54]	Platinum resistant or refractory recurrent ovarian cancer	Atezolizumab + Chemotherapy (PLD)	ORR, 13.3% (avelumab + CTx.) ORR, 4.2% (CTx.)
Phase II study conducted by Walsh et al. [61]	Platinum resistant recurrent ovarian cancer	Pembrolizumab + Chemotherapy (Gemcitabine and Cisplatin)	ORR, 60% PFS, 6.2 months OS, 11.3 months
Phase II study conducted by Wenham et al. (NCT02440425) [45]	Platinum resistant recurrent ovarian cancer	Pembrolizumab + Chemotherapy (Paclitaxel)	Ongoing
Phase III study conducted by Merck Sharp & Dohme LLC (NCT05116189) [46]	Platinum resistant recurrent ovarian cancer	Pembrolizumab + Chemotherapy (Paclitaxel or Docetaxel) ± Bevacizumab	Ongoing
Studies using combination therapy (ICI + chemotherapy + antiangiogenesis agent)			
Phase III study conducted by Moore et al. (NCT03038100) [62]	Advanced ovarian cancer	Atezolizumab + Bevacizumab + Chemotherapy (Paclitaxel and Carboplatin)	PFS, 19.5 months (PD-L1 negative) PFS, 20.8 months (PD-L1 positive)
Phase III study conducted by Kurtz et al. (NCT02891824) [47]	Platinum sensitive recurrent ovarian cancer	Atezolizumab + Bevacizumab + Chemotherapy (Platinum-based Chemotherapy)	Ongoing
Phage II study conducted by Zsiros et al. (NCT02853318) [63]	Platinum sensitive, resistant, or refractory ovarian cancer	Pembrolizumab + Bevacizumab + Oral Metronomic Cyclophosphamide	ORR, 47.5% (total) ORR, 66.0% (platinum sensitive) ORR, 43.3% (platinum resistant)
Phage Ib study conducted by Michels et al. (NCT03596281) [48]	Platinum resistant ovarian cancer	Pembrolizumab + Bevacizumab + Chemotherapy (Pegylated Liposomal Doxorubicin, PLD)	Ongoing
Studies using combination therapy (immunotherapy combination)			
Phase II study conducted by Zamarin et al. [52]	Persistent or recurrent ovarian cancer	Nivolumab + Ipilimumab	ORR, 31.4% PFS, 3.9 months

ICI, immune checkpoint inhibitor; CTx., chemotherapy; ORR, objective response rate; PFS, progression-free survival; PLD, PEGylated liposomal doxorubicin.

## 6. Use of ICIs in Cervical Cancer

Approximately 70% of cervical cancers worldwide are caused by persistent infections with human papillomavirus (HPV) 16 and 18 [64], and in contrast to other gynecological cancers, the cause of cervical cancer is relatively clear. The HPV vaccine was developed to prevent cervical cancer, and the United States Centers for Disease Control and Prevention analyzed the effect of HPV vaccination. The results demonstrated that the incidence of high-risk cervical cancer lesions, which can lead to cervical cancer caused by HPV, decreased by approximately 40%. Each year, approximately 36,500 men and women develop HPV-induced cancer in the United States, and HPV vaccination has confirmed the preventive effect of HPV-induced cancer in 33,700 individuals [65]. In a randomized, double-blind trial, a comparison of the vaccinated group with the quadrivalent vaccine against HPV and the placebo group showed that the incidence of cervical intra-epithelial neoplasia 2 (CIN2) and cervical intra-epithelial neoplasia (CIN3) was significantly lower in the vaccinated group than in the placebo group (vaccinated group, CIN2 147/6087, rate 0.9 vs. placebo group, CIN2 192/6080, rate 1.1; vaccinated group, CIN3 127/6087, rate 0.7 vs. placebo group, CIN3 8/6080, rate 0.9) [64].

**Figure 2:**
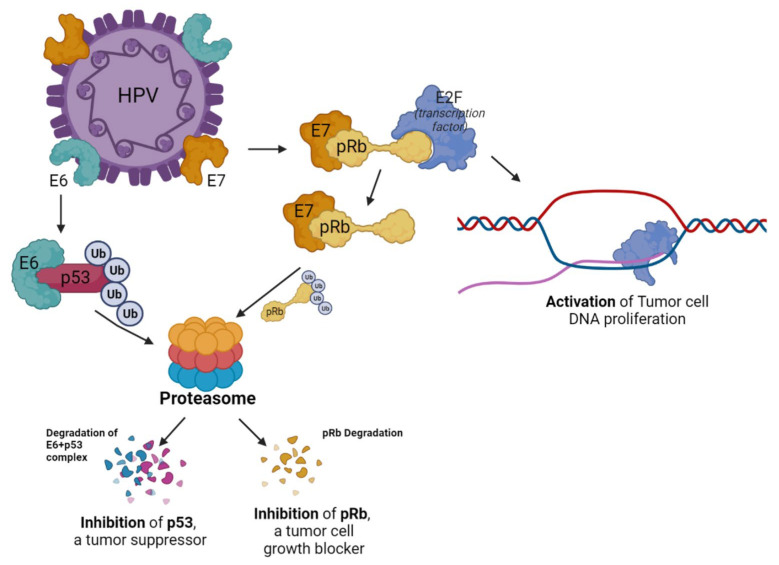
Mechanism between E6 and E7 of HPV and tumor cell proliferation.

HPV infection produces E6 and E7 proteins; E6 suppresses the tumor suppressor gene p53 and E7 suppresses the tumor suppressor gene Rb, causing the induction of cancer. E7 combines with APCs, such as dendritic cells, similar to non-inflammatory self-antigens, to create an immune-inhibited environment overall [66]. The mechanism by which E6 and E7 of HPV contribute to tumor cell proliferation is described in Figure 2. Considering that cervical cancer increases the risk of developing cervical cancer in immunocompromised patients with HIV, it can be suggested that immune system modulation therapy is helpful for the treatment of cervical cancer. The ORR observed after treatment with nivolumab in patients with cervical, vaginal, or vulva cancer was 20.8%, but the treatment results of nivolumab were not associated with HPV or PD-1 positivity [19]. In order to more clearly identify the relationship between the environment created by HPV and the results of ICI treatment, it is considered necessary to conduct additional studies, including not only cervical cancer, but also head and neck cancers, which are HPV-related cancers.

E6 of HPV inhibits the activity of p53, which serves as a tumor suppressor. E6 protein is ubiquitinated to form the E6/p53 complex, resulting in proteasomal degradation of E6/p53 complex. E2F, a transcription factor that contributes to the expression of tumor cell DNA, is in a state where its activity is suppressed in a complex with pRb. E7 of HPV interacts with the E2F and pRb complex to activate E2F and induce DNA proliferation in tumor cells. Additionally, pRb is ubiquitinated by E7 and degraded by proteasome.

There are many studies on the effect of ICIs treatment on cervical cancer, which are summarized in Table 3. The results of the phase Ib cohort study that confirmed the effect of pembrolizumab in PD-L1-positive advanced cervical cancer, also known as the KEYNOTE-028 trial, demonstrated an ORR of 17% (4 out of 24). Immune-related side effects after pembrolizumab treatment was observed in 75% of the patients; however, no Grade 4 treatment-related side effects or death were observed [67]. The phase 2 KEYNOTE-158 study was conducted to prove that the antitumor activity of pembrolizumab in patients with advanced cervical cancer demonstrated an ORR of 14.3% (complete response (CR) 2.6% and partial response (PR) 11.7%), and the ORR- of PD-L1-positive patients among all subjects was confirmed to be 16.0% [68]. These studies demonstrated the therapeutic effect of pembrolizumab in patients with PD-L1-positive cervical cancer and formed the basis for pembrolizumab to be approved by the FDA as a treatment for patients with progressive or recurrent cervical cancer with PD-L1 positivity. In the phase III study using cemiplimab, another PD-1 inhibitor, the OS in the cemiplimab group was 12 months, whereas the OS in the chemotherapy group was 8.5 months, a significant difference of HR 0.69 and *p*-value <0.001. Although there is a limitation that the PD-L1 test was not performed in all the groups using cemiplimab in this study, it was observed that the OS was 13.9 months for PD-L1 positivity and OS of 7.7 months for PD-L1 negativity, suggesting that the antitumor activity was better for PD-L1 positivity [69]. This suggests that other ICIs can be considered additional alternatives when treating patients with recurrent cervical cancer. A study that reported treatment results using atezolizumab, an anti-PD-L1 agent, and bevacizumab, an anti-angiogenesis agent, in advanced cervical cancer patients showed an ORR of 0%, which is significantly lower than results reported after using ICI and anti-angiogenesis drugs in endometrial cancer or ovarian cancer [70]. The aforementioned study conducted by Freideman et al. reported the following limitations. First, compared to KEYNOTE 158, which had a rate of adenocarcinoma and adenosquamous carcinoma in 6% of the subjects, in the phase II study, 45% of the subjects had adenocarcinoma and adenosquamous carcinoma, resulting in a low ORR. Second, all the study participants were patients who had been treated with bevacizumab before the study was conducted. Third, as only approximately 37.5% of the subjects were PD-L1 positive, the overall antitumor activity was inevitably reduced, with the majority being PD-L1-negative [70]. However, upon using the same atezolizumab and bevacizumab as combination therapy for renal cell carcinoma (RCC) and hepatocellular carcinoma (HCC) patients, the ORR of RCC patients was 37%, and that of HCC patients was 27%. Further research is needed to understand the complex interrelationship between the tumor microenvironment and immunotherapy in cervical cancer [71,72]. In addition, studies on combination therapy, such as durvalumab + tremelimumab, pembrolizumab + chemoradiation treatment (CCRT) and durvalumab + carbon ion radiotherapy (CIRT), are currently in progress for the treatment of advanced cervical cancer [73,74,75,76,77]. In line with conventional radiation treatment (RT), CIRT has the advantage of minimizing damage to the surrounding normal tissue and is effective in the treatment of locally existing lesions, owing to its high linear energy transfer [78]. A study comparing the results between the group treated with CIRT and the group treated with X-ray radiotherapy (XRT) has been reported for rectal cancer patients with locally recurrent lesions. The local recurrence rate of the group treated with CIRT was significantly lower with HR 0.17 and *p*-value = 0.002. The severe toxicity rate that appeared after treatment was also low, with HR 0.15 and *p*-value = 0.015 in the group treated with CIRT [79]. Although there are few studies reporting the use of CIRT in cervical cancer patients, there is a retrospective study comparing the standardized incidence ratios (SIRs) of cancer lesions post-photon RT and CIRT in cervical cancer patients. There was no statistically significant difference between the SIR result of 1.1 in the group subjected to CIRT and SIR results of 1.4 in the group subjected to photon RT (*p* = 0.268) [78]. A study on combination therapy by Okonogi et al. is in progress, and in this study, CIRT is used in patients with advanced cervical cancer (Table 3) [79].

**Table 3:** ICI monotherapy and combination therapy, including ICI used in cervical cancer.

Studies	Patient Subjects	Therapeutic Agent	Results
Studies using single ICI therapy			
Phase Ib study conducted by Frenel et al. [67]	PD-L1-positive advanced cervical cancer	Pembrolizumab	ORR, 17%
Phase II study conducted by Chung et al. [68]	PL-L1-positive advanced cervical cancer	Pembrolizumab	ORR, 14.3% (total) ORR, 16.0% (PD-L1+ patients)
Phase I/II study conducted by Lheureux et al. [80]	Recurrent cervical cancer	Ipilimumab (anti-CTLA-4 agent)	ORR, 2.9%
Phase I/II study conducted by Hollebecque et al. [81]	Recurrent cervical cancer	Nivolumab	ORR, 5%
Phase II study conducted by Santin et al. [82]	Persistent or Recurrent cervical cancer	Nivolumab	ORR, 4%
Phase III study conducted by Tewari et al. [69]	Recurrent cervical cancer	Cemiplimab	ORR 16.4% OS at 8.5 months
Phase I study conducted by Mayadev et al. [73]	Cervical cancer IB2/IIA with positive para-aortic LN only, Cervical cancer IIB/IIIB/IVA with positive LN following chemoradiation	Ipilimumab	Ongoing
Phase II study conducted by Lheureux et al. [74]	Metastatic or recurrent cervical caner	Ipilimumab	Ongoing
Phase II study conducted by Santin et al. [82]	Persistent or recurrent cervical cancer	Nivolumab	PFS at 6 months, 16% OS at 6 months, 78.4%
Studies using combination therapy (ICI + antiangiogenesis agent)			
Phase II study conducted by Friedman et al. [70]	Advanced cervical cancer	Atezolizumab + Bevacizumab	ORR, 0%
Studies using combination therapy (immunotherapy combination)			
Phase I study conducted by Callahan et al. [75]	Advanced cervical cancer	Durvalumab + Tremelimumab	Ongoing
Studies using combination therapy (ICI + CCRT)			
Phase II study conducted by Duska et al. [76]	Advanced cervical cancer in combination with chemoradiation	Pembrolizumab + CCRT	Ongoing
Studies using Combination therapy (ICI + CIRT)			
Phase Ib study conducted by Okonogi et al. [77]	Advanced cervical cancer	Durvalumab + CIRT	Ongoing

ICI, immune checkpoint inhibitor; CTLA-4, Cytotoxic T Lymphocyte Antigen-4; ORR, objective response rate; PFS, progression-free survival; OS, overall survival; CCRT, chemoradiation treatment; CIRT, carbon-ion radiotherapy.

## 7. irAE Mechanism

This part aimed to summarize the potential mechanisms of irAEs.

First, ICI therapy, such as via PD-1/PD-L1 and CTLA-4 inhibitors, inhibits the self-reactive T cell function, which can induce resistance to the immune process. PD-1 interacts with PD-L1 and PD-L2 in peripheral or tumor tissues to induce signals that suppress immune responses, primarily via immune response mediators, such as effector T cells, regulatory T cells, and B cells. PD-1 can also control central tolerance by adjusting the signaling thresholds during the T cell development process [14]. The PD-1 gene knockout mice experiments conducted by Nihimura et al. confirmed that immunological toxicity is induced within a limited range, such as organ-specific toxicity, rather than toxicity caused by lymphocyte production and development [18]. Second, treatment with PD-1 and PD-L1 inhibitors can directly or indirectly affect the human immune response, resulting in autoimmune disease. PD-1 signaling inhibits BCR signaling by dephosphorylating the major transducers in BCR signaling and recruiting SHP-2 [83]. Based on these mechanisms, Thibulet et al. reported an increase in B cell activation, B cell proliferation, and immunoglobulin secretion when interfering with the PD-1/PD-L1 signaling pathway [84]. These changes in B cells can create an environment that generates autoantibodies, and these results have been reported in several studies. Lupus-like proliferative arthritis and glomerulonephritis due to IgG3 deposition increased in PD-1 knockout mice [18], and the results of the follow-up conducted by collecting blood samples from melanoma patients who had undergone ICI treatment identified that new autoantibodies, such as anti-TPO and anti-TG, were generated in about 19% of patients who were negative for autoantibodies before ICI treatment [85]. ICI treatment can contribute to the activation of the autoimmune response by existing autoantibodies, and Toi et al. reported the incidence of irAEs in NSCLC patients who already had autoantibodies [86]. Osorio et al. also showed that autoantibodies are associated with hypothyroidism and pituitary inflammation induced by ICI treatment [87]. Third, as a result of damage to bystander cells during the antigen cross-presentation mechanism of the T cell-mediated immune response, the immune response in the body can target normal tissues and cause their aggression. After ICI treatment, the mechanism presented above can further increase the destruction of normal tissue, and cytotoxic T cells recognize the external antigen presented by APCs and then destroy cells with external antigens. During this process, non-transformed bystander cells can also be targeted and destroyed, and the resulting antigens are presented to T cells by the APC. Owing to this, T cells perform autoimmune activities that recognize normal tissues as targets and attack them [88]. Johnson et al. reported that normal muscle-specific antigens were presented as antigens by APCs in patients who underwent combination ICI treatment using both ipilimumab and nivolumab, resulting in an immune response that attacked tumor cells, as well as normal myocardium [88]. In addition, cases of vitiligo and autoimmune pigmentary disorders have been reported as a result of autoimmune reactions targeting normal melanocytes in melanoma patients who received ICI treatment via a similar mechanism [89]. Fourth, changes in the composition of the intestinal bacteria are related to irAEs that occur after ICI treatment. Enterobacteriaceae play a critical role in intestinal health, as well as strengthening the intestinal epithelial barrier and protecting the intestinal environment from pathogens [90]. A comparison of the intestinal bacteria analysis and ICI treatment outcomes in NSCLC patients treated with nivolumab revealed that the ICI treatment outcome was good in the intestinal environment rich in *Alistipes putreinis*, *Bifidobacterium longum*, and *Prevotella copri*. However, patients rich in *Ruminococcus* (unclassified) in the intestinal environment showed a poor response to ICI therapy [91]. IrAE is also associated with enterobacteria, and Liu et al. compared fecal samples from various lung cancer patients treated with ICI and confirmed that immune-related diarrhea occurred more frequently when *Veillonella* enteric bacteria were abundant and *Parabacteroides* and *Phascolarctobacterium* were scarce [92]. Conversely, in patients with diarrhea after ipilimumab treatment for melanoma, a relatively high amount of *Phascolarctobacterium* was detected, unlike with lung cancer [93]. The composition of the intestinal environment may change, depending on the type of cancer being treated with ICI, and this aspect requires further study.

## 8. Organ-Specific Toxicities Induced by ICI Treatment and Their Management

**Figure 3:**
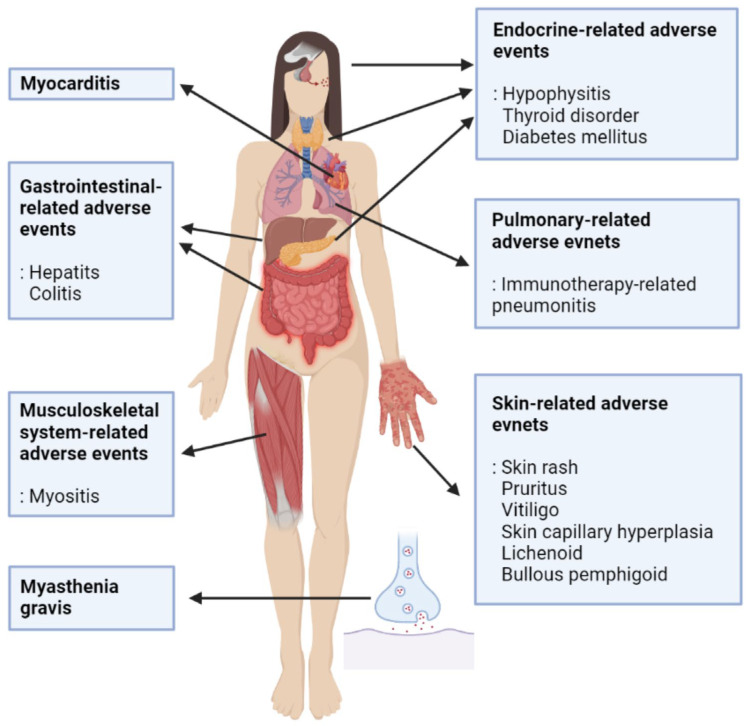
The organ-specific toxicities that can be induced by ICI therapy.

The organ-specific toxicites that can be induced by ICI therapy are as follows (Figure 3).

### 8.1. Skin-Related Adverse Events

The most common irAE after ICI treatment is skin disease. Dermatologic toxicities tend to occur in approximately 30% of patients treated with ICI, and skin lesions include rash, pruritus, vitiligo, skin capillary hyperplasia, lichenoid, and bullous pemphigoid [19]. In a study comparing the degree of dermatological side effects after pembrolizumab and nivolumab use, the relative risk (RR) of skin lesions after the use of pembrolizumab and nivolumab was similar (RR = 2.6 and RR = 2.5, respectively) [94]. A comparison of the occurrence of skin lesions caused by the PD-1 inhibitor pembrolizumab and the PD-L1 inhibitor atezolizumab showed that skin adverse events were generally induced after the second treatment with pembrolizumab. The incidence of skin lesions was higher in cases where the PD-1 inhibitor was used than in the cases where the PD-L1 inhibitor was used (11–31% and 7–19%, respectively) [7]. Although more prospective studies are needed, it has been reported that patients with CR/PR after cancer treatment tend to have more adverse skin events than those with SD [95].

Dermatologically-related adverse events were graded according to severity, from Grade 1 to Grade 4. Grade 1 refers to a case in which skin macules and papules account for <10% of body surface area (BSA), and this does not interfere with daily life. Topical steroids can be applied to skin lesions or oral antihistamines can be administered if necessary. Immunotherapy need not be discontinued because of Grade 1 skin adverse events [19]. When the skin macules and papules account for approximately 10–30% of BSA, the lesions are considered as Grade 2, and they slightly interfere with daily life. Considering that there is a possibility of deteriorated renal and liver functions, it is necessary to conduct numerical routine laboratory tests, such as the liver function test (LFT), BUN, and creatine during the work-up process [19]. Management may be performed using oral prednisone 0.5–1 mg/kg/day, an oral antihistamine agent, and ICI therapy should be stopped if symptoms do not improve after 12 weeks [7]. If the symptoms continue to improve, oral steroids should be gradually tapered over an interval of one month or more, and ICIs therapy can be resumed when the skin lesion has improved to Grade 1 or less [19,96]. Grade 3 is when there are enough sequelae to limit life, and Grade 4 is when there are enough sequelae to threaten life. Grades 3 and 4 should be treated as severe events, and appropriate management should be performed. When skin macules and papules exceed 30% of BSA and Steven–Johnson syndrome is present, it is classified as Grade 4. In these cases, open skin ulcers or wet peeling may occur [19]. To differentiate impetigo, staphylococcal scalded skin syndrome (4S), acute generalized exanthematous pustulosis (AGEP), and other diseases that cause bullous lesions, it is necessary to perform a skin biopsy in the vesicular skin area at Grades 3 and 4 [97]. In the case of serious sequelae, such as Grade 3 or Grade 4, ICI immunotherapy should be stopped first, and a high-potency topical steroid should be applied to the affected skin lesion [19]. Oral prednisone 0.5–1 mg/kg/day can be used. If there is no effect, the oral prednisone dose can be increased to 2 mg/kg/day, and in patients with severe pruritus, GABA agonists, such as gabapentin and pregabalin, can be used [7].

### 8.2. Gastrointestinal-Related Adverse Events

#### 8.2.1. Hepatitis

Hepatitis is a sequela that occurs less frequently in ICI-treated patients, and it has been reported that hepatitis occurs with a probability of approximately 5% when using PD-1 inhibitors. The incidence of hepatitis is 30% in combination therapy using ipilimumab and nivolumab [98]. There was a tendency for hepatic-related side effects to occur approximately 8–14 weeks after ICIs therapy was started, and hepatic adverse events could be classified into Grade 1 to Grade 4, according to severity [19]. Considering that most patients who have undergone ICI therapy are not aware of the side effects related to liver dysfunction without symptoms, it is critical to compare the LFT results obtained before starting immunotherapy and before each immunotherapy to determine whether liver enzyme dysfunction is progressing. ICIs therapy may cause hepatitis; therefore, it is essential to identify causes, such as alcohol, viral infection, side effects caused by other drugs, and liver dysfunction caused by cancer progression, before arriving at a conclusion [98]. In addition, cases of hepatomegaly, periportal edema, and periportal lymphadenopathy were reported approximately 12 weeks after using ipilimumab; therefore, it is necessary to perform imaging tests, such as CT and MRI, and perform liver biopsies if necessary [99].

In Grade 1 of hepatic-related side effect AST/ALT levels are ≤2.5 × ULN (upper limit of normal range) or total bilirubin level is ≤1.5 × ULN of liver enzyme dysfunction. The liver function test should be performed, and progress should be made until the enzyme level is normalized. If it does not improve and demonstrates a worsening trend, ongoing ICIs therapy needs to be stopped [19,98]. Cases where 5 × LLN (lower limit of normal range) ≤ AST/ALT ≤ 5 × ULN or 1.5 × LLN ≤ total bilirubin ≤3 × ULN belong to Grade 2. In this case, ICIs therapy should be temporarily stopped, and oral steroid management and LFT should be monitored and followed up. If there is a trend of improvement below Grade 1, ICIs therapy may be resumed again, and the oral steroid preparation used gradually tapered over a period of more than one month [19,98]. In Grades 3–4, with AST/ALT ≥ 5 × ULN or total bilirubin ≥ 3 × ULN, the possibility of liver enzyme dysfunction caused by cancer progression cannot be ruled out; therefore, after an imaging test, liver biopsies should be performed if necessary. In such cases, immunotherapy should be stopped, IV steroids should be used, and daily LFT monitoring should be performed. If symptoms and LFT levels improve, the IV steroid used should be tapered over a period of more than one month [19,98]. If symptoms do not improve after 3–5 days despite IV steroids, additional immunosuppressive agents, such as mycophenolate mofetil or infliximab, need to be used [20].

#### 8.2.2. Colitis

Diarrhea is one of the most common adverse events of ICIs therapy. On average, it tends to occur after approximately two or three ICIs treatments. Diarrhea symptoms were more common in about 30–40% of patients who used ipilimumab, and the incidence of severe diarrhea, such as Grades 3–4, was observed in about 1% of patients who used ipilimumab. Incidence of diarrhea in patients who used anti-PD-1 agent or anti-PD-L1 agent was about 1–2%, and the incidence was higher when ipilimumab was used than when PD-1 inhibitors or PD-L1 inhibitors were used [100]. In addition, in the case of combination therapy using ipilimumab and a PD-1 inhibitor, colitis occurred with a 44% probability, and in the case of single therapy using an anti-CTLA-4 agent or an anti-PD-1 agent, colitis occurred with 20% probability. It has been confirmed that there is an increase in the tendency for colitis to occur when using combination therapy [98].

Grade 1 colitis adverse events refer to diarrhea occurring less than four times a day; immunotherapy can be continued, and symptomatic treatment can be performed. If symptoms worsen, even after three days, despite treatment, Grades 2–4 management should be implemented [19]. The case of four to six diarrhea symptoms per day belongs to Grade 2, which may be accompanied by abdominal pain, mucus, or bloody stools. It is recommended to conduct a stool test to check whether diarrhea is caused by an infectious disease and hold immunotherapy until the symptoms improve. Symptomatic treatment is performed using oral steroid preparations and antidiarrheal drugs. If symptoms improve, immunotherapy is resumed, and oral steroid agents are gradually tapered over a period of one month or more [19,98]. In Grades 3–4, the patient may have diarrhea more than seven times a day and display severe abdominal pain and peritoneal signs. After stopping ICIs therapy, additional imaging tests and, if necessary, endoscopy should be performed to differentiate abdominal pain and diarrhea caused by other causes. If diarrhea due to an adverse event induced after immunotherapy is confirmed, the patient should be hospitalized, treated with intravenous steroids, and followed up. If the patient’s symptoms do not improve after three days, additional immunosuppressive agents, such as infliximab, should be considered [19,98].

### 8.3. Endocrine-Related Adverse Events

Among the endocrine-related adverse events induced after immunotherapy, the most common ones include acute hypophysitis and thyroid disease or abnormal thyroid function levels. Endocrine-related adverse effects include hypophysitis, Type 1 diabetes mellitus, and thyroid dysfunction, such as hypothyroidism and hyperthyroidism [98]. However, as symptoms due to these endocrine abnormalities appear non-specific, such as fatigue, headache, and nausea, it is difficult to understand whether the immune-related endocrine adverse effects are induced by the symptoms alone. Therefore, it is important to conduct tests, such as thyroid function tests and adrenal function tests, for adrenocorticotropic hormone (ACTH), cortisol, glucose, and HbA1c before immunotherapy. Before administering ICIs therapy to patients, it is also important to determine whether there are symptoms suggestive of endocrinopathies.

#### 8.3.1. Hypophysitis

Hypophysitis has various symptoms that depend on the specific hormone deficiency induced. When hypothyroidism occurs, symptoms such as weight gain and vulnerability to cold occur, and when secondary hypogonadism is present, symptoms such as amenorrhea and erectile dysfunction can be induced. It can range from asymptomatic or mid-symptoms to life-threatening levels when progressing to acute panhypopituitarism [101]. Therefore, if hypophysitis is suspected, the pituitary hormone and target tissue hormone levels should be measured to determine which organ’s hormone is deficient, and it is critical to adjust the hormone balance by supplementing the insufficient hormones. In addition, high-dose steroids should be administered as an acute treatment. Generally, methylprednisolone 1–2 mg/kg per day IV is used for three to five days, followed by prednisone 1–2 mg/kg per day, and gradually tapered over a period of four weeks or more. Another option is the use of dexamethasone as an alternative steroid treatment regimen. The method of using 4 mg of dexamethasone every 6 h is performed for one week, and gradually tapered by 0.5 mg/day [102]. It is essential to understand that gradually tapering the steroid dose is critical in preventing adrenal crisis. The results of meta-analysis conducted by Barroso-Sousa et al. reported that the prevalence of hypophysitis after pembrolizumab and atezolizumab was 0.9% and <0.1%, respectively, and when ipilimumab was used, the prevalence of hypophysitis was 3.2%. The prevalence of hypophysitis was higher in the case of using ipilimumab than in the case of using pembrolizumab and atezolizumab [103]. In a study comparing the incidence of hypophysitis according to the dose of ipilimumab, it was reported that the prevalence of hypophysitis was significantly higher in the group receiving >3 mg/kg ipilimumab (9–7% incidence) than in the group using <3 mg/kg ipilimumab (1.8–3.3% incidence) [103]. Although the mechanism underlying the relationship between the use of ICI agents and the induction of hypophysitis has not been clearly elucidated, IgG-1 autoantibodies targeting FSH, TSH, and ACTH were observed in patients who developed an inflammatory reaction in the pituitary gland after CTLA-4 inhibitor treatment. Additionally, antibody-mediated type II hypersensitivity to ectopic CTLA-4 protein in the pituitary gland is activated in patients with hypophysitis. This suggests that autoimmune antibodies and hypersensitive immune-related reactions may be related to the mechanism of hypophysitis [101].

#### 8.3.2. Thyroid Disorder

ICIs therapy causes thyroid toxicity, resulting in thyroid dysfunctions, such as hypothyroidism or hyperthyroidism. Although the mechanism underlying the association between ICIs and thyroid toxicity is not clear, it has been reported that CTLA-4 inhibitors are associated with CTLA-4 gene polymorphisms and the high prevalence of Graves’ disease and Hashimoto’s thyroiditis [101]. PD-1 and PD-L1 inhibitors accumulate anti-thyroglobulin (thyroglobulin), anti-thyroid peroxidase (TPO) antibodies, and T cells through T cell-and B cell-mediated immune reactions to destroy thyroid tissue in the same manner as Hashimoto thyroiditis [104]. Therefore, thyroid dysfunction induced by ICIs may be related to an autoimmune mechanism.

Among ICIs therapy, after ipilimumab treatment, the incidence of primary hypothyroidism was 3.8% and that of secondary hypothyroidism was 7.6%. After PD-1 and PD-L1 inhibitor treatment, the incidence of primary hypothyroidism was 3.9–7.0%, and that of secondary hypothyroidism was <1%, showing various prevalence rates [104]. Studies have analyzed the occurrence of immune-related reactions, such as thyroid dysfunction, after immunotherapy. According to a retrospective review conducted by Ryder et al., thyroid toxicity occurred approximately five months to three years after treatment with a CTLA-4 inhibitor [105]. In another retrospective study conducted on melanoma patients who had been treated with pembrolizumab, it was confirmed that thyroid toxicity was induced approximately six weeks after using a PD-1 inhibitor [106]. Thyroid toxicity is usually very similar to thyroiditis and occurs mainly in subclinical or mild hypothyroidism. Thyroid function tests revealed low thyroid-stimulating hormone (TSH), elevated free T4, anti-TPO, and anti-TG antibodies, and normal thyroid stimulation immunoglobulins. There tends to be a sequence of thyroid toxicities, followed by hypothyroidism [107]. The thyroid glands in ICI-treated patients observed on CT and PET-CT appeared to be homogenous and hyperdense. However, in the case of thyroid toxicity due to immunotherapy, enlarged, hypoenhanced, or hypodense thyroid glands are generally observed. In the case of immunotherapy-related hypothyroidism, a hypodense and diminished thyroid gland is observed [101]. Therefore, when thyroid function abnormalities are suspected in patients under ICI therapy, additional imaging tests, such as ultrasound, CT, and PET-CT, as well as thyroid function tests, may help diagnose immune-related thyroid dysfunction. Immunotherapy-related primary hypothyroidism can be treated by supplementing the thyroid hormone with levothyroxine. It is usually administered at 1.6 µg/kg per day, and symptoms improve after about several weeks; however, as elevated TSH is maintained for a while, it is recommended to follow up and control TSH after approximately 4–8 weeks of starting the treatment. Subclinical hypothyroidism does not require oral therapy when symptoms are absent [102]. The thyroid hormone balance should be controlled using anti-thyroid drugs during hyperthyroidism, and when atrial fibrillation and tachycardia are accompanied by hyperthyroidism, a beta-blocker should also be used to control the symptoms [101]. Graves’ ophthalmopathy has been reported in patients treated with ipilimumab alone or in combination with bevacizumab [108]. Typical symptoms of Graves’ ophthalmopathy include proptosis and periorbital edema, and systemic symptoms include double vision, blurred vision, ocular pain, and intolerance to bright light. In cases of systematic and severe symptoms, it is necessary to use a high-dose glucocorticoid treatment to monitor whether symptoms improve [102].

#### 8.3.3. Diabetes Mellitus

Among diabetes, type 1 diabetes mellitus (DM) is a disease caused by the autoimmune destruction of pancreatic beta cells. Even when ICIs therapy is performed, type 1 DM may occur during the immune-related reaction, and the mechanism underlying this is not clear. Glutamic acid decarboxylase 65 (GAD65) autoantibodies are commonly observed in patients with type 1 DM and patients with diabetes after ICIs treatment, and studies have reported that PD-1 expression in T cells is commonly reduced in patients with type 1 DM and ICI-related DM [109,110]. Based on this, it is hypothesized that an autoimmune reaction targeting pancreatic beta cells will induce inappropriate T cell activation and abnormal blood glucose control, resulting in adverse events, such as diabetes, after immunotherapy.

DM occurred in approximately 0.2–0.9% of patients treated with PD-1/PD-L1 inhibitor, and there was a 1.5% probability of DM after combination therapy using CTLA-4 inhibitor and PD-1 inhibitor [101]. Symptoms appear at various time intervals for each patient, such as diabetes occurring less than a month or a year after starting ICI therapy, and blood HbA1c and glucose levels also tend to vary from patient-to-patient [101].

Considering that immunotherapy-related DM is caused by autoimmune reactions, such as type 1 DM, symptoms progress in a manner similar to those of patients with type 1 DM. Type 1 DM patients have ketogenic hyperglycemia, which develops into diabetic ketoacidosis when left untreated [102]. Consequently, symptoms such as polydipsia, polyuria, weight loss, and abdominal pain may appear. Therefore, patients receiving ICIs therapy should check whether these symptoms develop and check the GAD65, anti-insulin, anti-islet cell, C-peptide, and insulin levels to differentiate between type 1 DM and type 2 DM [98]. If type 1 DM is diagnosed, appropriate blood glucose control should be performed using insulin, and if necessary, patients should be managed with the help of an endocrinologist.

### 8.4. Pulmonary-Related Adverse Events

The most common immunotherapy-related pulmonary reaction is pneumonitis, which tends to occur between seven days and 19.2 months after ICIs therapy [111]. Comparing the incidence of pneumonitis after treatment with a PD-1 inhibitor and PD-L1 inhibitor, the overall incidence of pneumonitis and the incidence of severe Grade pneumonitis were higher upon treatment with PD-1 inhibitors than with PD-L1 inhibitors (total pneumonitis incidence, PD-1 inhibitor vs. PD-L1 inhibitor, 3.6% vs. 1.3%; severe pneumonitis incidence, PD-1 inhibitor vs. PD-L1 inhibitor, 1.1% vs. 0.4%) [112]. The incidence of pneumonitis, when treated with combination therapy with ICIs was higher than when treated with ICI monotherapy (pneumonitis incidence, combination therapy vs. monotherapy, 10% vs. 3%) [111].

ICI-related pneumonitis can cause a wide range of symptoms, from asymptomatic cases to life-threatening cases, as well as interfering with daily life. Therefore, even when the patient does not complain of respiratory symptoms, it is necessary to perform a basic imaging examination, such as chest radiography, before ICIs therapy to check for abnormalities. If respiratory symptoms and abnormal lesions are suspected on chest X-ray examination, a chest CT should be performed. With CT, ICI-related pneumonitis can be observed in the following five types of lesions: cryptogenic organizing pneumonia (COP), ground-glass opacities (GGO), interstitial, hypersensitivity, and pneumonitis not otherwise specified [111]. For such drug-induced pneumonitis, a biopsy should be performed on the chest lesion area to differentiate whether the lesion is caused by cancer metastasis or other causes, rather than ICIs. In the case of pneumonitis induced by an anti-PD-1/PD-L1 agent, biopsy results may be helpful in diagnosis because one or more of the following pathological findings are observed: cellular interstitial pneumonitis, organizing pneumonia, diffuse alveolar damage, granuloma formation, and eosinophils [111].

If there are no symptoms and Grade 1 with mild lesions is observed in imaging, chest CT should be performed within four weeks to work-up, and ICIs therapy can be continued, while checking for respiratory symptoms for three days [7,19,98]. For Grade 2 patients with respiratory symptoms that affect daily life, immunotherapy is first administered, and then the daily symptoms are checked. To confirm *Pneumococcus* and *Legionella* infection, nasal swab, sputum culture, blood culture, and urine antigen tests are performed. This procedure should be performed in Grade 2, Grade 3, and Grade 4 events. Additionally, bronchoscopy and bronchoalveolar lavage should be performed, and prophylactic antibiotics may be used when bacterial infection cannot be excluded. In Grade 2 events, prednisone/methylprednisolone 1–2 mg/kg/day is administered orally or intravenously for treatment, and when symptoms are relieved to Grade 1 within three days, immunotherapy is resumed, and the steroid is gradually tapered at intervals of four weeks or more. However, when symptoms do not improve after three days, immunotherapy should be stopped [7,19,98]. In Grades 3 and 4, there are severe respiratory symptoms that are life-threatening, and additional oxygen supply is required due to hypoxia. As mentioned previously, a chest lesion biopsy can be performed to check whether the abnormalities in the respiratory system are due to lesions caused by cancer metastasis or other diseases; when the patient is diagnosed with ICI-related pneumonitis, immunotherapy should be stopped, and methylprednisolone 1–2 mg/kg/day and prophylactic antibiotic treatment should be administered via IV. If the symptoms improve, steroids should be gradually tapered over a period of six weeks or more; however, if the symptoms worsen within 2 days, additional immunosuppressive treatment should be considered [7,19,98].

### 8.5. Musculoskeletal System-Related Adverse Events

Among patients treated with ICIs, patients experiencing musculoskeletal pain due to irAEs in the musculoskeletal system are often observed. However, it is critical, even though difficult, to differentiate immuno-related skeletal muscular adverse events because of the possibility of pain caused by cancer itself or because severe pain caused by metastasis of the cancer lesion to the bone cannot be excluded. In a retrospective study, immune-related musculoskeletal adverse reactions occurred more frequently in the group using nivolumab monotherapy than in the group using pembrolizumab and durvalumab, as well as in the group using nivolumab and ipilimumab. The musculoskeletal symptoms tend to appear approximately 48 weeks after the start of treatment [113]. Creatinine kinase (CK) levels, troponin levels, anti-acetylcholine receptor antibodies, and myositis-associated antibodies should be checked for abnormalities, and laboratory results, such as CK, troponin, anti-acetylcholine receptor antibodies, and myositis-associated antibodies levels, are not consistent. In the study conducted by Tout et al., the blood CK level was elevated to 2668 U/L in all patients who complained of immune-related skeletal muscle symptoms, and the more severe the pain, the higher the CK level. Troponin T levels were elevated in seven out of nine patients, and anti-acetylcholine receptor antibodies and myositis-associated antibodies were negative in all patients [113]. In the case of immuno-related myositis, when muscle biopsy is performed, necrotic myofibers, hyalinized necrotic myofibers, and myophagocytes are observed generally or focally, and immunochemically, CD68 and CD8 are positive [113].

In cases of mild painful myositis, immunotherapy can be continued, and a serial lab follow-up, such as checking the CK level, is performed. For moderate or severe pain, immunotherapy should be performed, prednisone 1–2 mg/kg/day should be used until symptoms improve, and muscle biopsy should be performed, if necessary [7].

### 8.6. Myasthenia Gravis

Myasthenia gravis is another rare adverse event induced after ICIs therapy. Myasthenia gravis is a basic clinical symptom of muscle weakness and muscle fatigue, and the symptoms are mild in the morning, but tend to worsen in the afternoon and temporarily improve after rest, such as sleeping. Muscle weakness, which is mainly controlled by the cranial nerve, may occur and cause symptoms such as drooping eyelids, impaired vision, weakness in facial muscles, weakness in chewing, difficulty in pronunciation, difficulty in swallowing, and difficulty in breathing. An analysis of 23 patients who developed myasthenia gravis after ICIs therapy showed that myasthenia gravis symptoms started within approximately six weeks of starting immunotherapy [114]. Another study reported that myasthenia gravis occurred more frequently when a PD-1 inhibitor was used than when a PD-L1 inhibitor was used [115].

In cases of moderate to severe grade myasthenia gravis, ICIs therapy should be permanently discontinued, and treatment with oral pyridostigmine 30 mg three times a day should be started. The dose needs to be increased to a maximum of 120 mg four times a day, while observing the symptoms. Alternatively, there is a treatment regimen that starts with 20 mg daily low-dose oral pyridostigmine and gradually increases the dose within a range that does not exceed 100 mg/day. In the case of considerably severe symptoms, a regimen in which methylprednisolone 1–2 mg/kg/day and rituximab 375 mg/m^2^ weekly are used together and can be administered four times [7]. In a cohort study of 496 patients treated with nivolumab, Guillain–Barré syndrome, myasthenia gravis, and polyneuropathy occurred as side effects. The patient was treated with corticosteroids and anti-inflammatory drugs, and their symptoms improved. However, despite concurrent treatment with immunosuppressive drugs, neurological side effects did not improve and persisted [116]. In other words, prompt recognition and treatment of adverse events in patients can be helpful in improving the symptoms, as well as in reducing the sequelae that may persist thereafter.

### 8.7. Myocarditis

The occurrence of myocarditis is rare among the adverse events induced after immunotherapy. Although rare, there are reports of a 75-year-old NSCLC patient who developed myocarditis three days after the ninth cycle of nivolumab treatment and a 68-year-old NSCLC patient who developed myocarditis after receiving nivolumab treatment [117,118]. Although the mechanisms underlying the association between immunotherapy and the development of myocarditis are unclear, Wang et al. reported that T cell infiltration was activated in PD-1 deficient myocardium in an experiment using PD-1 deficient mice, resulting in severe heart disease [119]. This suggests that PD-1 plays a critical role in protecting heart tissue from T cell-mediated immune reactions. In addition, the myocardial tissue biopsy of a patient who developed myocarditis after treatment with PD-1 inhibitor revealed the same CD8-positive T cell observed in the tumor biopsy, thus supporting the previous claim [120]. Symptoms of myocarditis include acute chest pain and dyspnea, and abnormal findings, such as ventricular tachycardia, ectopic ventricular beats, and abnormal ST-segment, can be observed via electrocardiography.

Although there is no typical treatment for immunotherapy-related myocarditis, a heart failure therapy regimen using angiotensin-converting enzyme inhibitors, beta-blockers, diuretics, and prednisolone 1 mg/kg/day is preferred. Additionally, immunosuppressive agents, such as anti-thymocyte globulin, infliximab, and mycophenolate, can be used for treatment [7,117].

## 9. Conclusions

With the development of modern medical technology, the mortality rate of gynecological cancers, such as cervical, endometrial, and ovarian cancers, has improved. Despite surgical treatment, multiple chemotherapies, and radiation therapy, the available treatments for progressing gynecological cancers are inevitably limited. In this context, ICI therapy, which induces anti-tumor activity by activating tumor-related immune reactions inhibited by cancer, can be considered a treatment for patients with advanced gynecological cancer. Many studies have reported the response rate after administering ICI monotherapy and combination therapy, including ICI, for the treatment of gynecological cancer, and other studies are currently in progress. Although it is well-known that PD-1 and PD-L1 inhibitors have high reactivity in PD-1-positive gynecological cancer patients and ICI inhibitor reactivity is high in endometrial cancer in MSI-H or d-MMR environments, in order to effectively apply such immunotherapy in the treatment of gynecological cancer, it is crucial to understand the mechanisms of action of anti-PD-1, anti-PD-L1, and anti-CTLA-4 agents and how immunotherapy is performed in gynecological cancers. In addition, it is necessary to be aware of the various immune-related adverse events that may be induced after immunotherapy and to implement the appropriate treatment accordingly. This review is relevant because it summarizes the information necessary for gynecologists to use ICI therapy in treating gynecological cancers.
